# Search for differentially methylated regions in ancient and modern genomes

**DOI:** 10.18699/VJGB-23-95

**Published:** 2023-12

**Authors:** D.D. Borodko, S.V. Zhenilo, F.S. Sharko

**Affiliations:** Federal Research Center “Fundamentals of Biotechnology” of the Russian Academy of Sciences, Moscow, Russia; Federal Research Center “Fundamentals of Biotechnology” of the Russian Academy of Sciences, Moscow, Russia; Federal Research Center “Fundamentals of Biotechnology” of the Russian Academy of Sciences, Moscow, Russia

**Keywords:** ancient DNA, methylation, epigenetics, DamMet, DMR, древняя ДНК, метилирование, эпигенетика, DamMet, ДМР

## Abstract

Currently, active research is focused on investigating the mechanisms that regulate the development of various pathologies and their evolutionary dynamics. Epigenetic mechanisms, such as DNA methylation, play a significant role in evolutionary processes, as their changes have a faster impact on the phenotype compared to mutagenesis. In this study, we attempted to develop an algorithm for identifying differentially methylated regions associated with metabolic syndrome, which have undergone methylation changes in humans during the transition from a hunter-gatherer to a sedentary lifestyle. The application of existing whole-genome bisulfite sequencing methods is limited for ancient samples due to their low quality and fragmentation, and the approach to obtaining DNA methylation profiles differs significantly between ancient hunter-gatherer samples and modern tissues. In this study, we validated DamMet, an algorithm for reconstructing ancient methylomes. Application of DamMet to Neanderthal and Denisovan genomes showed a moderate level of correlation with previously published methylation profiles and demonstrated an underestimation of methylation levels in the reconstructed profiles by an average of 15–20 %. Additionally, we developed a new Python-based algorithm that allows for the comparison of methylomes in ancient and modern samples, despite the absence of methylation profiles in modern bone tissue within the context of obesity. This analysis involves a two-step data processing approach, where the first step involves the identification and filtration of tissue-specific methylation regions, and the second step focuses on the direct search for differentially methylated regions in specific areas associated with the researcher’s target condition. By applying this algorithm to test data, we identified 38 differentially methylated regions associated with obesity, the majority of which were located in promoter regions. The pipeline demonstrated sufficient efficiency in detecting these regions. These results confirm the feasibility of reconstructing DNA methylation profiles in ancient samples and comparing them with modern methylomes. Furthermore, possibilities for further methodological development and the implementation of a new step for studying differentially methylated positions associated with evolutionary processes are discussed.

## Abstract (ru)

В настоящее время активно исследуются механизмы, регулирующие развитие различных патологий и их эволюционную динамику. Эпигенетические механизмы, такие как метилирование, играют значимую роль в эволюционных процессах, поскольку их изменения гораздо быстрее отражаются на фенотипе, чем результаты мутагенеза. В данном исследовании мы предприняли попытку разработать алгоритм для выявления дифференциально метилированных областей, связанных с метаболическим синдромом, которые изменили свое метилирование у человека при переходе от охоты и собирательства к оседлой жизни. Применение существующих методов полногеномного бисульфитного секвенирования ограничено для древних образцов из-за их низкого качества и фрагментации, и подход к получению профилей метилирования охотников-собирателей значительно отличается от подходов, используемых для современных тканей. В этой работе мы валидировали DamMet – алгоритм, реконструирующий древние метиломы. Применение DamMet к геномам неандертальца и денисовца показало средний уровень корреляции с профилями метилирования, опубликованными ранее, а также продемонстрировало занижение уровня метилирования реконструированных профилей в среднем на 15–20 %. Также мы разработали новый алгоритм на языке Python, позволяющий сравнивать метиломы в древних и современных образцах, несмотря на отсутствие профилей метилирования современных образцов костной ткани в контексте ожирения. Такой анализ подразумевает двухступенчатую обработку данных, где на первом этапе происходит идентификация тканеспецифичных областей метилирования и их фильтрация, а на втором этапе осуществляется непосредственно поиск дифференциально метилированных регионов в заданных областях, ассоциированных с интересующим исследователя заболеванием. В результате использования алгоритма на тестовых данных мы обнаружили 38 дифференциально метилированных регионов, ассоциированных с ожирением, большая часть которых принадлежала промоторным областям, и разработанный пайплайн показал достаточную эффективность в их поиске. Эти результаты подтверждают возможность восстановления профилей метилирования в древних образцах и их сравнения с современными метиломами. Также обсуждаются возможности дальнейшего развития методологии и внедрения нового шага, позволяющего изучать дифференциально метилированные позиции, связанные с эволюционными процессами.

## Introduction

Lately, increasing attention is being paid to the study of mechanisms regulating the development of various pathologies and their evolutionary dynamics (Briggs et al., 2009a; Niiranen et al., 2022). Epigenetic mechanisms, such as methylation, play a particularly important role in this process since they are capable of inducing phenotypic changes much faster than conventional mutagenesis processes (Jablonka, Raz, 2009; Feinberg, Irizarry, 2010; Zhur et al., 2021). The main goal of this study was to identify differentially methylated regions (DMRs) associated with metabolic syndrome, which could potentially serve as targets for epigenetic therapy of metabolic syndrome.

Nowadays, scientists are often hindered from conducting evolutionary research due to the lack of suitable methods for comparing DNA profiles of ancient and modern samples. Laboratory protocols used to obtain these profiles significantly differ from one another, each having its peculiarities and errors. Ancient DNA (aDNA) is often found in a fragmented state, and over time, natural molecule degradation and spontaneous deamination of nitrogenous bases occur, limiting the availability of high-quality data (Briggs et al., 2007, 2009b). To address this issue, a specific sample processing protocol was developed, which uses uracil-DNA glycosylase (UDG) and endonuclease combination (known as USER-treatment) to facilitate the extraction of methylation profiles and enhance their distinguishability (Briggs et al., 2010). Additionally, several programs have been developed that allow the calculation of methylation levels in ancient samples, the sequences of which were sequenced using the USER treatment (Gokhman et al., 2014; Orlando et al., 2015; Hanghøj et al., 2019).

At present, two methylation reconstruction algorithms tailored for ancient samples are available, characterized by their command-line functionality and user-friendliness. The antecedent algorithm, epiPALEOMIX, draws its foundation from the initial historical approach to methylation reconstruction, as first elucidated by D. Gokhman in 2014. EpiPALEOMIX encompasses diverse modules, among which the MethylMap module stands out, permitting users to derive methylation levels in regions that can be defined by the user (Hanghøj et al., 2016). However, this limitation is inherent to its usage; the user is required to have an understanding of the particular regions associated with the condition under study. The outcome of this algorithm is the calculated count of deaminated methylated cytosines in the CpG context and the corresponding coverage, representing their ratio, thereby denoting the methylation level at the particular genomic position. In contrast, the DamMet algorithm exhibits greater versatility. Unlike epiPALEOMIX, it is designed for whole-genome investigations. Furthermore, DamMet can calculate deamination levels in both methylated and unmethylated CpGs at each read position, thus employing a model that most accurately characterizes the deamination of cytosines in aDNA fragments as a random process (Hanghøj et al., 2019).

Regarding the handling of modern tissue samples, wholegenome bisulfite sequencing (WGBS) is the prevalent method for investigating DNA methylation (Olova et al., 2018; Suzuki et al., 2018). Several methods are available for reconstructing methylation from samples sequenced using this technology (Clark et al., 1994; Bock et al., 2005), with the most well-known being Bismark, BoostMe, and WGBStools. Currently, the Bismark algorithm is the most frequently used for preprocessing WGBS data. This involves the mapping of reads to the converted reference genome, followed by the quantification of methylated and unmethylated cytosines at each genomic position (Krueger, Andrews, 2011). Similar to many read-count-based methods, this approach is not wellsuited for overcoming the challenge of low sample coverage, a common occurrence in cases involving low-quality samples or single-cell experiments. To address this concern, machine learning-based algorithms like DeepCPG and BoostMe have been created.

DeepCPG is a deep learning neural network-based algorithm designed to predict the methylation states of lowcoverage sites and uncover motifs associated with changes in methylation levels and intercellular variability (Angermueller et al., 2017). This tool is primarily utilized to enhance the quality of data from single-cell experiments. BoostMe, which is based on a machine learning approach, addresses this issue during the genome preprocessing stage by employing imputation (Zou et al., 2018). The XGBoost gradient boosting technique employed in this tool amalgamates data from multiple samples (more than 3) to rectify missing methylation levels in contemporary tissue samples. This enables the utilization of low-coverage genome samples for methylation reconstruction. Additionally, a notable feature of BoostMe is its capacity to restore not only the state of a given CpG site (methylated/ unmethylated) but also its methylation level. WGBStools, comprising a collection of methods developed in the context of the modern tissue methylation atlas project, is utilized for a highly efficient representation of mapped reads, statistical analysis, and visualization of data ranging from small genomic segments to entire chromosomal loci (https://github.com/ nloyfer/wgbs_tools).

However, despite the variety of methylation reconstruction algorithms available, the application of WGBS technology to aDNA samples is limited. This limitation arises from the requirement for a high concentration of well-purified DNA for bisulfite conversion. Additionally, the bisulfite conversion process leads to DNA fragmentation, further compromising the quality of aDNA, which is already significantly fragmented due to degradation (Gu et al., 2011). Therefore, methylation level calculation algorithms commonly used for modern samples cannot be employed for the reconstruction of methylation profiles in ancient individuals. Consequently, our focus has been on developing a novel algorithm that enables the comparison of methylomes in ancient and modern samples, considering the lack of available bone tissue samples for conducting whole-genome bisulfite sequencing in the context of obesity.

## Materials and methods

Sample selection. For our analysis, we curated a dataset from the NCBI GEO database, consisting of 11 ancient genomes and 12 modern methylation profiles obtained using Whole Genome Bisulfite Sequencing (WGBS) methods. When selecting the ancient samples, particular attention was given to the age of the samples, library preparation strategy, and genome coverage. We exclusively included samples that underwent prior USER treatment, were dated to be at least 3,000 years Before the Common Era (BCE), and had a minimum coverage of 5x. The complete genomes of ancient samples were sequenced with USER treatment, except for samples Vi33 and PES001 (Peschanitsa), which were not subjected to UDG treatment before sequencing (Table 1).

**Table 1.**
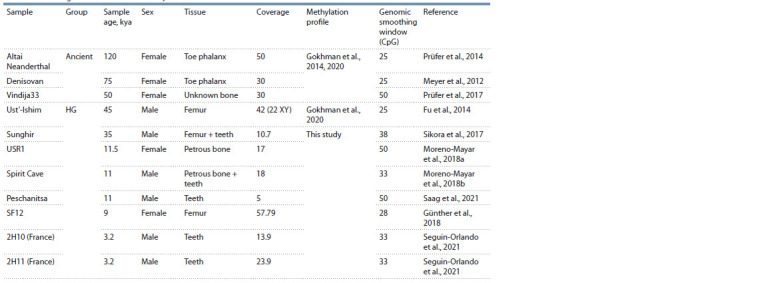
Ancient genomes selected for analysis Note. Smoothing window – a parameter for averaging deamination levels in the subsequent analysis stage. HG – hunter-gatherers.

The selection of the 12 contemporary samples (Loyfer et al., 2023) was based on the mesodermal origin of the tissues used for library preparation, in conjunction with the utilization of whole-genome bisulfite sequencing. Additional information about these samples is presented in Table 2.

**Table 2.**
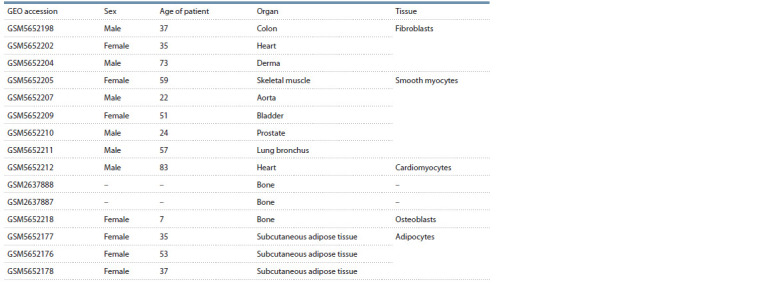
Contemporary genomes used for identifying tissue-specific methylated regions and DMRs

Ancient genomes preprocessing. The ancient genomes were obtained from ftp server in bam format along with their corresponding indices. As per previous studies (Ohm et al., 2010; Gokhman et al., 2014), it is well-recognized that UDG treatment is not sufficiently effective at the DNA termini. To ensure precise aDNA analysis, we employed the trimBam utility to trim two nucleotides from both the 3ʹ and 5ʹ ends of sequences (Gansauge, Meyer, 2013; Jun et al., 2015). It’s important to note that for the Vi33 and PES001 samples, this trimming procedure was omitted due to the absence of UDG treatment during library preparation. Moreover, we applied Trimmomatic (Bolger et al., 2014) for the filtration of sample reads based on criteria such as average quality and length. In our subsequent analysis, only sequences that aligned with the CRCh37 (hg19) assembly and exhibited an average quality score exceeding 20, as well as a minimum length of 25 base pairs, were retained for further investigation.

Reconstruction of DNA methylation profiles in ancient humans. To reconstruct the methylation profiles of ancient samples, we utilized the DamMet software (Hanghøj et al., 2019). The pipeline consisted of three main stages: the filtration of single-nucleotide variants, the calculation of deamination levels for each read position, and the estimation of methylation levels

The single-nucleotide variant (SNV) calling was performed using the GATK HaplotypeCaller v4.3.0.0 (Poplin et al., 2017). SNVs with coverage of less than 5 and quality less than 30 were filtered out. Additionally, variants were filtered when they exhibited homozygosity for the alternative allele or more than two alternative alleles when the position contained a cytosine. This stage followed the recommendations of the DamMet algorithm author, as described in Hanghøj et al., 2019, and supplementary materials provided therein.

Subsequently, methylation levels were reconstructed, excluding the identified variants.

DamMet estDEAM -b <bam-file> -r <fasta-file> -c <chromosome> -M <expected-average-methylation> -O <out-file-prefix> -E <vcf-to-exclude> -L 25 -P 50 -q 20 -Q 20

Subsequently, we determined the methylation levels based on the identified deamination levels at positions with both methylated and demethylated cytosines. The genomic window size for each sample is indicated in the respective column of Table 1 and was selected through empirical evaluation

DamMet estF -b <bam-file -r <fasta-file>-c <chromosome> -M <expected-average-methylation> -O <out-file-prefix -N <genomic-window-sizein- CpGs>

The acquired methylation profiles were additionally subjected to smoothing using a Python script that applied a moving average with a smoothing window size of 25 CpG sites

Validation of the reconstructed methylomes. The comparison of Neanderthal, Denisovan, and Ust-Ishim hunter-gatherer methylomes obtained in the previous stage was conducted using the R programming language. We employed packages like ggplot, psych, corr.test, and the tidyverse family for data preprocessing, correlation analysis, and graph generation.

Identification of tissue-specific methylated regions. We designed a Python script for the identification of regions exhibiting relatively consistent methylation levels across all mesodermal tissues. This script takes the methylation values obtained using the Bismark algorithm (Krueger, Andrews, 2011) after aligning the aforementioned samples as input. It conducts a per-position comparison of methylation values through ANOVA to detect variations within three tissue groups (fibroblasts, myocytes, osteoblasts) and exclude positions showing statistically significant differential methylation (p < 0.05) from both ancient bone and modern adipocyte methylation profiles

DMR identification. The prepared methylation profiles of hunter-gatherers (HG) and modern individuals were compared using the ANOVA method, similar to the tissue-specific methylation search. In the first iteration, the samples were divided into three groups: hunter-gatherer bone samples, healthy individuals’ adipocytes, and obese patients’ adipocytes. CpG sites with a significance level of p < 0.05 were selected for subsequent analysis using the Tukey post hoc test. ACpG site was considered differentially methylated if the methylation change was significant (p < 0.05) when comparing HG bones to adipocytes of obese individuals and not significant when comparing HG bones to controls

In the second iteration, we modified the grouping: all samples were bone samples, and the groups represented samples of different ages (anatomically ancient humans, hunter-gatherers, and modern humans). Comparisons were made only in regions associated with obesity to reduce the computational load. To aggregate the obtained differentially methylated sites into regions, we used the combined-pvalues software (https://github.com/brentp/combined-pvalues), which is based on the Stouffer–Liptak multiple testing correction method (Pedersen et al., 2012). The methylation change status was determined by comparing the mean methylation values in the regions between groups

## Results

**Fig. 1.**
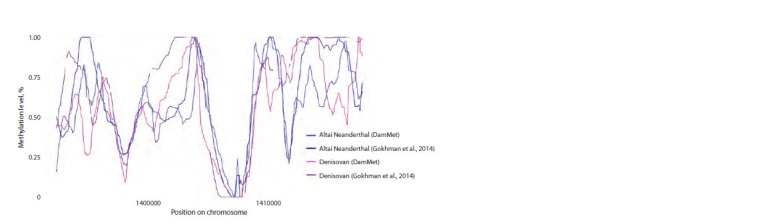
Comparison of the methylation profiles of a Denisovan and a Neanderthal reconstructed by DamMet and published by D. Gokhman In focus: a demethylated CpG island at chr1:1406845–1407821.

In this study, we reconstructed 11 DNA methylation profiles of ancient humans using the DamMet tool. Firstly, we needed to develop a pipeline that would allow us to reconstruct methylomes with high precision. For this purpose, we used the genomes of Neanderthals and Denisovans, which had undergone UDG treatment, as input data for the pipeline. Profiles for these organisms had previously been published (Gokhman et al., 2014, 2020), enabling us to validate the pipeline. We found that our calculated methylation levels were, on average, 15–20 % lower than those previously published, but overall, the methylation profiles were similar (Fig. 1). The correlation coefficients for methylation profiles in both cases were over 85 %: rDenisovan = 0.87, rNeanderthal = 0.9 ( p < 0.05).

As we had several samples that didn’t undergo USER treatment during library preparation, we also aimed to confirm whether DamMet could reconstruct methylation profiles without this step. To address this, we selected sampleVi33, for which sequences both with and without USER treatment were publicly available. The pipeline parameters were consistent for these analyses, ensuring uniform conditions for reconstructing methylomes from both libraries.

**Fig. 2.**
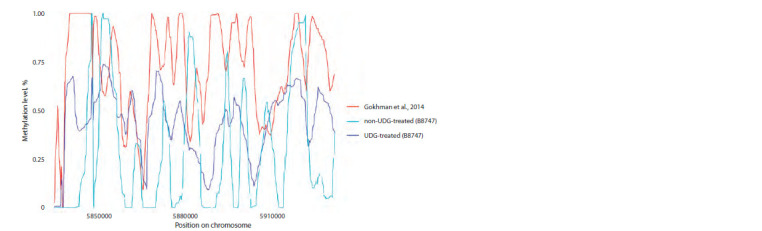
Comparison of methylation levels on a region of chromosome 2 in sample Vi33, in the presence and absence of USER treatment during library preparation, with previously published profiles by D. Gokhman. Methylation levels of all samples were smoothed using a 25 CpG moving average.

Our findings revealed that the methylation profile obtained in the presence of USER treatment showed an average correlation of 0.57 with the profile calculated by D. Gokhman, as depicted in Figure 2. In contrast, the methylome obtained without any treatment displayed a weak correlation (r = 0.14) with the published profile. Notably, the methylation patterns primarily matched in demethylated CpG islands, irrespective of whether we applied subsequent smoothing using a moving average.

**Fig. 3.**
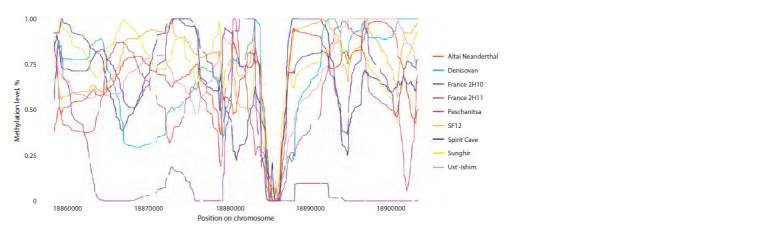
Methylation profiles of hunter-gatherers reconstructed using DamMet The region of extensive demethylation corresponds to the CpG island at chr21:18884807–18886111 (GRCh37 hg19). exon exon/intron intergenic intron promoter Fig. 4. Percentage distribution of DMRs in various genomic regions. 23 % 60 % 10 % 5 %

Next, we processed eight genomes of hunter-gatherers using our pipeline, for which methylation profiles had not been reconstructed previously (see Table 1). The resulting profiles generally exhibited a similar methylation pattern to other ancient methylomes, including complete demethylation of some CpG islands (Fig. 3), resembling the profile of the previously reconstructed Ust-Ishim hunter-gatherer (Gokhman et al., 2020). Even though sample PES001 was not subjected to USER treatment during library preparation, our obtained methylation profile exhibited overall trends similar to other hunter-gatherer profiles and thus was not excluded from further analysis.

According to the authors of the method, the reconstructed methylation profiles using DamMet can be used for direct comparison with modern data. However, methylation can vary between cells of different origins, so direct comparisons should be limited to methylation profiles obtained from the same tissues. To the best of our knowledge, there has been no sequencing of bone tissues in the context of obesity. Therefore, for the final comparison, we selected samples from subcutaneous and visceral adipocyte tissues, which exhibit similar methylation patterns. However, these patterns may significantly differ from those observed in bones and other mesodermal tissues. As a result, we developed a Python script that performs a search for differentially methylated positions in mesodermal tissues and excludes them from further analysis. The script is based on dispersion analysis in three groups, followed by pairwise comparisons and multiple testing corrections. The mesodermal tissue samples were divided into groups according to tissue type: fibroblasts, muscle cells, and osteoblasts. In total, about 26.5 million CpG positions were analyzed, with approximately 206,000 showing differential methylation in at least one group, while more than 26 million did not exhibit significant differences.

**Fig. 4.**
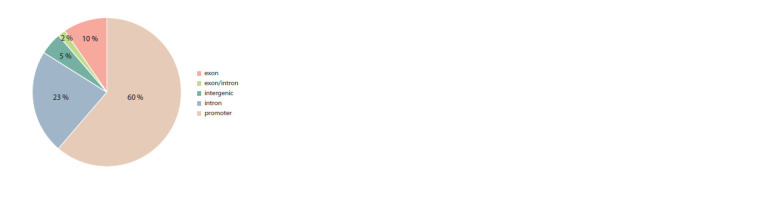
Percentage distribution of DMRs in various genomic regions

We conducted a search for Differentially Methylated Regions (DMRs) in modern bone tissue samples, but focused our search on only 642 regions that had been previously associated with differential methylation in the context of obesity, as reported in the literature. In this case, we performed a perposition ANOVA analysis for groups of ancient individuals, hunter-gatherers, and modern individuals (bone tissue), with prior filtering of non-tissue-specific CpG sites. We identified 38 DMRs, where the overlap with the aforementioned 642 regions included more than 20 CpG sites. As depicted in Figure 4, approximately 60 % of these DMRs are located in gene promoter regions, 35 % are within gene body regions, and only 5 % of the DMRs are situated in intergenic regions. Notably, 94 % of these DMRs exhibit hypermethylation, potentially leading to the suppression of gene expression, particularly in genes associated with obesity.

Supplementary data and source code

The methylation profiles of ancient humans and the Python scripts used for the analysis in this study are available in the GitHub repository: https://github.com/bor-d/ancDMR

## Conclusion

There are currently several methods available for reconstructing methylation profiles of ancient organisms, with epiPALEOMIX (Hanghøj et al., 2016) and DamMet (Hanghøj et al., 2019) being the two most commonly used ones. While both of these methods are known for their significant accuracy, their performance is often constrained by the quality of ancient DNA samples. In our study, we opted to utilize the DamMet method due to its versatility, specifically its capacity to compare the reconstructed methylation values with profiles generated using alternative sequencing technologies. However, during the validation of our pipeline, we observed notable discrepancies between the methylation values obtained with DamMet and those previously published by D. Gokhman, in both 2014 and 2020. The developers of DamMet acknowledge that their tool tends to yield lower methylation values in comparison to profiles generated using epiPALEOMIX, which does not account for factors such as single nucleotide variants (SNVs), sequencing errors, and the demethylation of unmethylated cytosines. This was evident in our reconstruction of Neanderthal and Denisovan profiles. Nonetheless, our analysis indicated a positive correlation between the methylation values reconstructed by DamMet and the previously published data. This reaffirms the tool’s effectiveness in reconstructing previously uncharacterized methylation profiles, which can then be used for subsequent comparisons with modern methylomes.

In a demonstration of the pipeline we had devised, we attempted to identify DMRs within the genomic profiles of hunter-gatherers and contemporary humans, specifically in the context of obesity. We identified 38 regions, with approximately two-thirds of them located in promoter regions. This observation implies a plausible association between alterations in methylation patterns within these promoters and the regulation of gene expression. Certainly, the well-defined procedural stages within our pipeline effectively tackle potential hurdles researchers might face. This is especially valuable when dealing with situations where there is a lack of published methylation profiles related to the specific tissues of interest. These steps help reduce the likelihood of false-positive DMRs due to tissue-specificity

When utilizing this pipeline to investigate DMRs related to different medical conditions, researchers are advised to conduct a thorough review of relevant scientific literature. This exploratory endeavour should ultimately lead to the discovery of regions where methylation patterns are inherently connected to the specific condition being studied. However, it is imperative to underscore that despite the explicit precautions taken, including the exclusion of tissue-specific regions and stringent filtering in the context of disease-associated regions, the investigation of DMRs may still encompass CpG sites, the methylation profiles of which underwent alterations during the evolutionary transition from archaic humans (Homo sapiens neanderthalensis) to contemporary Homo sapiens sapiens.

## Conflict of interest

The authors declare no conflict of interest.
